# Students' Perceptions of a Blended Learning Environment to Promote Critical Thinking

**DOI:** 10.3389/fpsyg.2021.696845

**Published:** 2021-06-25

**Authors:** Dan Lu

**Affiliations:** School of Foreign Languages, Northeast Normal University, Changchun, China

**Keywords:** students' perceptions, blended learning environment, critical thinking, design, survey

## Abstract

Critical thinking is considered as one of the indispensable skills that must be possessed by the citizens of modern society, and its cultivation with blended learning has drawn much attention from researchers and practitioners. This study proposed the construction of a blended learning environment, where the pedagogical, social, and technical design was directed to fostering critical thinking. The purpose of the study was to find out students' perceptions of the learning environment concerning its design and its influence on their critical thinking. Adopting the mixed method, the study used questionnaire and interview as the instruments for data collection. The analysis of the data revealed that the students generally held positive perceptions of the environment, and they believed that the blended learning environment could help promote their critical thinking in different aspects.

## Introduction

The development of critical thinking has drawn attention of the education ministries and institutions of different levels in countries all over the world. In the last two decades, researchers and practitioners have been exploring the ways to integrate critical thinking cultivation into the instruction of different disciplines, proposing strategies and interventions to promote critical thinking, among which blended learning has been widely recognized (e.g., Van Gelder and Bulker, 2000; Gilbert and Dabbagh, 2005; Yukawa, 2006). Blended learning is proposed as focusing on optimizing achievement of learning objectives by applying the “right” personal learning technologies to the “right” person at the “right” time and “right” place (Singh, 2003). A blended learning environment, integrating the advantages of the e-learning method and traditional method, is believed to be more effective than a face-to-face or online learning environment alone (Kim and Bonk, 2006; Watson, 2008; Yen and Lee, 2011). Studies have been conducted to construct blended learning environments to improve students' critical thinking. Most of them, however, adopted standardized tests or coding schemes to examine the effectiveness of the learning environments on students' critical thinking (Chou et al., 2018), paying less attention to students' perceptions and attitudes. Therefore, the purpose of the current study is to address this gap.

### Critical Thinking

There are a vast number of definitions of critical thinking in the literature (e.g., Paul, 1992; Ennis, 1996; Fisher and Scriven, 1997). Despite the emphasis on different aspects, the core of critical thinking entails taking charge of one's thinking to improve it. Paul and Elder's definition and model of critical thinking were adopted in the study. According to Elder and Paul (1994), critical thinking refers to “the ability of individuals to take charge of their own thinking and develop appropriate criteria and standards for analyzing their own thinking” (p. 34). They proposed that critical thinking is composed of three dimensions: elements of thinking, intellectual standards, and intellectual traits. People demonstrate critical thinking when they use intellectual standards (clarity, precision, accuracy, importance, relevance, sufficiency, logic, fairness, breadth, depth) to measure elements of thinking (purposes, assumptions, questions, points of view, information, implications, concepts, inferences) (Paul and Elder, 1999).

### Critical Thinking Cultivation With Information Communication Technology Tools

Studies applying ICT tools to cultivate critical thinking have been increasingly emerging in the literature. The systematic review conducted by Chou et al. (2018) analyzed and reported the trends and features of critical thinking studies with ICT tools. According to the findings of the review, the most often used tools include online discussion (e.g., Cheong and Cheung, 2008), coding or game design or Wikibooks creation (e.g., Yang and Chang, 2013), and concept or argument maps (e.g., Rosen and Tager, 2014). As for the method involved, the studies adopted both quantitative and qualitative research methods (e.g., Shamir et al., 2008; Yang, 2008; Yang and Chou, 2008; Butchart et al., 2009; de Leng et al., 2009; Yeh, 2009). Data from various measurements revealed overall positive results of using ICT tools in critical thinking cultivation (e.g., Yang, 2008; Allaire, 2015; Shin et al., 2015; Huang et al., 2017). The findings of the systematic review showed that the critical thinking-embedded activities using ICT tools were more effective than face-to-face activities in developing students' critical thinking (Guiller et al., 2008; Adam and Manson, 2014; Eftekhari et al., 2016). However, students' prescriptions of the learning design or critical thinking development have not been fully addressed in the literature.

### Blended Learning Environment

The concept of blended learning has been defined by several researchers and scholars. For instance, Singh and Reed (2001) defined blended learning as a learning program where more than one delivery mode is being used to optimize the learning outcome and cost of program delivery. According to Thorne (2003), blended learning is a way of “meeting the challenges of tailoring learning and development to the needs of individuals by integrating the innovative and technological advances offered by online learning with the interaction and participation offered in the best of traditional learning” (p. 2). The above definitions indicate that blended learning can combine the advantages of both traditional face-to-face learning and e-learning and avoid the drawbacks of the two learning modes. The effectiveness of blended learning has been demonstrated by many studies, for example, the findings of a meta-analysis have shown that blended learning brings more positive impact on students learning than online and face-to-face learning (BatdÄ, 2014). Despite the merits of blended learning itself, the effectiveness is determined by the proper design. How to achieve the equilibrium between e-learning and face-to-face modes is crucial to the success of the blended learning environment (Osguthorpe and Graham, 2003).

This study applied the PST model developed by Wang (2008) as the framework for the environment design. As Kirschner et al. (2004) pointed out, an educational system is a unique combination of pedagogical, social, and technological components. PST model thus consists of three key components: pedagogy, social interaction, and technology. According to Wang (2008), the pedagogical design involves the selection of appropriate content, activities, and the way to use the resources; the social design refers to the construction of a safe and comfortable environment where learners can share and communicate; the technical design provides learners with a technical space of availability, easy access and attractiveness. In any learning environment, the three components play different roles. The technical design offers a basic condition for pedagogical and social design, while the pedagogical and social design is considered as the most important factor that influences the effectiveness of learning (Wang, 2008).

### Perceptions of Blended Learning Environment

It has been acknowledged that students' perceptions and satisfaction are important for determining the quality of blended learning environment (Naaj et al., 2012). Studies have been conducted to examine students' views regarding a blended learning environment and factors influencing it. For example, Bendania (2011) study found that students hold positive attitudes toward the blended learning environment and the influencing factors mainly include experience, confidence, enjoyment, usefulness, intention to use, motivation, and whether students had ICT skills. The positive view was also reported in the study done by Akkoyunlu and Yilmaz (2006), and it was found to be closely related to students' participation in the online discussion forum. Findings from other studies (e.g., Dziuban et al., 2006; Owston et al., 2006) also revealed students' positive attitudes toward the blended learning environment, and the satisfaction could be attributed to features like flexibility, convenience, reduced travel time, and face-to-face interaction. Some studies, however, reported some negative perceptions of the blended learning environment. For example, the results of the study of Smyth et al. (2012) showed that the delayed feedback from the teacher and poor connectivity of the internet were perceived as major drawbacks of the environment. In another study conducted by Stracke (2007), lack of reciprocity between traditional and online modes, no use of printed books for reading and writing, and use of the computer as a medium of instruction was considered as major reasons for students withdraw from the blended course. These findings indicate that students' negative attitudes toward the blended learning environment mainly come from the inadequate design (Sagarra and Zapata, 2008).

The review of the above studies indicates that applying ICT tools to cultivate critical thinking has gained much popularity and produced positive results. Few studies, however, focus on students' perceptions of a learning environment designed to promote critical thinking despite the fact that many studies have been conducted to explore students' perceptions of a blended learning environment in general. Therefore, the purpose of the current research is to investigate students' perceptions of a blended learning environment with the orientation of critical thinking development.

## Research Design

### Research Questions

By adopting the mixed method, this study aims to answer the following two questions:

1. What are students' perceptions of the blended learning environment to promote critical thinking?
2. How do students perceive the impact of the blended learning environment on the development of their critical thinking?

### Context and Participants

The study was carried out in the course of Practical English Writing which is a branch of the comprehensive English course for first-year non-English majors at a Normal University in mainland China. The 6-week course adopted a mixed learning mode of classroom face-to-face and online learning. The face-to-face class ran once a week and each class was 90 min. The e-learning tasks were assigned either before or after the class. Six independent learning centers with networked computers were available for students to use and the whole campus was covered with Wi-Fi signal.

The participants of the study involved a total of 90 non-English major students (33 males and 57 females) aging from 18 to 20 in 2020. The students were allocated into classes of Level A after the placement test of English proficiency, which means their English was about higher intermediate level. Adopting the International Critical Thinking Reading and Writing Test (Paul and Elder, 2006), which was developed from Paul and Elders' thinking model, the study assessed students' critical thinking level at the beginning of the course and found that the students' overall critical thinking level was at the lower medium level. But their information literacy level was sufficient to cope with the online platform and the software in the blended learning environment. Before the implementation of the course, the instructor informed the students about the study, and the consent forms were signed by the students.

### Environment Design

For the learning environment to achieve the purpose of developing learners' critical thinking, its structural components should be designed to provide favorable conditions for critical thinking cultivation. A systematic review conducted by Lu (2018) has identified a series of favoring conditions that could promote the students' critical thinking, which include (a) critical thinking as one of the teaching objectives, (b) tasks involving the operation of ideas, (c) authentic context, (d) rich and diversified resources, (e) interaction and collaboration, (f) scaffolding and guidance, (g) communicative tools. These conditions were mapped to the design of the components of the PST learning environment model and the designing strategies were generalized from the instruction practice to guide the detailed design of the environmental components.

#### Pedagogical Design

In terms of the pedagogical design, the thinking skills that can be cultivated were first decided according to the particular learning content. Aiming at promoting the thinking skills, the learning tasks which mostly introduced problems in the “real” context and involve the operation of ideas were designed. Furthermore, rich and diversified resources were provided to the students. The specific strategies of pedagogical design are listed in Table 1.

**Table 1:** Strategies for pedagogical design.

**Guiding principles**	**Strategies**
Critical thinking skills as learning objectives	Introducing the concepts and frameworks of critical thinking to students
	Informing students of critical thinking as learning objectives
Operation of ideas	Including tasks of writing, discussion, and evaluation
	Choosing topics that induce collisions of ideas
Authentic context	Providing sufficient details
	Creating interesting situations
Rich and diverse resources	Collecting information from different media and perspectives
	Providing students with relevant websites and searching engines

When designing the learning objectives of the activities, the basic concepts and frameworks of critical thinking were introduced to the students, making them aware of its meaning and significace. Furthermore, students were informed of the thinking skills targeted and their importance. When students associated the thinking skills with the tasks, they would try to use the skills to accomplish them.

In order to enable tasks to involve more operations of ideas, writing, discussion, and evaluation activities were given the priority to provide more opportunities for students to communicate with each other and reflect upon their ideas. Besides, the topics of these activities were chosen to induce more collision of ideas. For example, in learning to write complaint letters, students were assigned the roles of customers who made the complaints and the managers who responded to the complaints. In such an activity, students could realize the existence of different perspectives and think more adequately and deeply.

The creation of a relatively real context drew on the following two strategies: One is to provide sufficient details. In the case of the job application writing, details such as the information about the potential employer were provided to the students so that they could consider themselves as “real” potential employees. The other strategy is to create interesting situations. The contexts described were usually attractive to the students, which helped arouse students' interest in completing the tasks.

With the purpose of collecting sufficient and diversified resources, both traditional and online media were included. Since the materials in the textbook are rather limited, the relevant online resources would make complementation for students to have sufficient resources to deal with. To meet the multi-angle nature of resources, the information collected came from different positions and perspectives. For instance, the students were introduced to the websites both for job hunting and recruitment so that they could read information from the perspectives of both employers and potential employees. To help students conduct resource searches by themselves, online resources such as the Online Writing Lab of Purdue University were presented to them to conduct searches. The search was usually directed by a clear question or a problem, and students needed to accurately identify the target source. Some search engines were also introduced to the students, enabling them to compare and select the relevant resources. Students needed to first define what their search objectives were, then assess the search and query results one by one, and finally synthesize the resources to make a reasonable decision.

#### Social Design

With the purpose of cultivating students' critical thinking in the environment, interactions and collaborations of different types were stressed in the design (see Table 2). Furthermore, the scaffold and guidance from the teacher and the peer were designed to provide support to the students.

**Table 2:** Strategies for social design.

**Guiding principles**	**Strategies**
Interaction and collaboration	Grouping students according to the features of the tasks Designing interaction of different types
	Creating various opportunities for students to communicate with the teacher
Scaffolding and guidance	Emphasizing the process of thinking Giving full play to the role of peers
	Creating “democratic” atmosphere Setting up an encouragement system

In designing interaction and collaboration-rich community, the strategies were applied to target both student-student and student-teacher communities. In terms of student-student community, students were grouped according to their levels and the requirements of the activities. Specifically, in a demanding task, students of different academic levels were grouped to ensure the implementation. In a relatively free discussion, students were grouped according to their own will so that they could feel more comfortable sharing their ideas. Also, various types of interactions such as information exchange, discussion, debate were designed. With the change of partners, roles, and tasks, different critical thinking skills were trained. As for the student-teacher community, the student-teacher communication was facilitated through various forms of teacher-student interaction, such as teachers' feedback, office hour, and communications on Tencent QQ, which were necessary to keep students on the right track of developing thinking skills. With various opportunities of communicating with the teacher, students would not feel powerless or frustrated when facing difficult tasks, thus ensuring the achievement of the learning objectives.

Four strategies were employed when designing the scaffolding and guidance. First, the process of thinking was highlighted. When the focus fell on critical thinking processes such as establishing viewpoints, making assumptions, and evaluating information, students had examples to follow when they conducted these activities independently. Second, the role of peers was given full play. In many cases, the demonstration of peers was more direct and effective for the students to develop critical thinking skills. Third, the teacher consciously created a “democratic” classroom and online atmosphere, where students could express their opinions without fearing judgment from the “authority” or other people. Fourth, the teacher established awarding incentives to encourage students to take the initiative to meet challenges and develop thinking. For example, if one student's feedback to others' work was deeper and more thorough, the instructor gave the student more marks and demonstrated the work to the whole class with their permission.

#### Technological Design

Moodle (Modular Object-Oriented Dynamic Learning Environment) was the main platform of the e-learning environment. A composition reviewing and grading software TRP (Teaching Resources Platform) was also used to facilitate teachers' grading of the compositions. TRP mainly focuses on the mistakes related to language and grammar, which could help direct teachers' attention to the composition's structure, logic, coherence, and other aspects. In addition, Tencent QQ, a social networking software frequently used by students, was selected to send messages and notices to students.

As shown in Table 3, both synchronous and asynchronous instruments were applied to provide sufficient communication among students in designing communicative tools. When designing the synchronous instruments, the instructor used the Tencent QQ, which could conveniently support the simultaneous real-time communication between learners and encourage group members to fully communicate with each other. The discussion board of Moodle was used as asynchronous tools, and sufficient time was given to the students to respond to other people's opinions or solve problems. The students could use the time to find information, consult others and translate complex ideas into words.

**Table 3:** Strategies for technological design.

**Guiding principles**	**Strategies**
Communicative tools	Choosing the most convenient tools for synchronous communication
	Leaving enough time for asynchronous communication

### Research Instruments

#### Learning Environment Questionnaire

The questionnaire adapted from the Web-Based Learning Environment Instrument (WEBLEI) was used to elicit the information of students' perception of the learning environment. The original WEBLEI questionnaire was first created and subsequently modified by Chang and Fisher for investigating online learning environments in University settings. The primary purpose of the questionnaire was to capture “students' perception of web-based learning environments” (Chang and Fisher, 2003, p. 9). The questions in the WEBLEI questionnaire are able to cover the three elements of the PST learning environment model. The researcher modified the questionnaire according to the context of the current study. The Cronbach alpha coefficients indicated the acceptable reliability of the modified questionnaire (see Table 4).

**Table 4:** Cronbach alpha coefficients for modified WEBLEI.

**Scales**	**Cronbach's alpha**	**N of items**
Pedagogical design	0.764	7
Social design	0.805	7
Technical design	0.884	6
Total	0.901	20

#### Interviews

In order to explore students' perceived improvement of critical thinking and the in-depth reasons behind students' perceptions of the learning environment and critical thinking instruction, interviews were conducted after the administration of the adapted WEBLEI questionnaire. Eight students were randomly chosen and invited to the interview one by one. The interviews lasted about 30 min and were audio-recorded with the participants' approval.

### Data Analysis

Both quantitative and qualitative data were collected for this study. In terms of quantitative analysis, descriptive statistics were used to describe the means, standard deviations. As for qualitative data, the recordings of the interviews were transcribed for content analysis. The content about the perceptions of the environment was categorized with the outline of the learning environment components. Regarding the development of students' critical thinking, the “elements of thinking” from Paul and Elder's thinking model formed the framework for data analysis. The relevant script was examined and coded according to the framework by the researcher and her collegue to generalize the aspects of critical thinking improvement.

## Results and Discussion

### Students' Perceptions of the Environment

#### Students' Perception of the Pedagogical Design

The means and standard deviation scores of students' perception of the pedagogical design are listed in Table 5. The overall mean score was 3.86 (SD = 0.79), suggesting that students were generally satisfied with the pedagogical design. Item 1 (M = 3.98, SD = 0.80) (The learning objectives are clearly stated), Item 4 (*M* = 3.93, SD = 0.83) (Expectations of assignments are clearly stated), and Item 5 (M = 4, SD = 1.00) (Activities are planned carefully) got particularly high scores, which indicates that students were aware of the careful design of the activities, content, and context.

**Table 5:** Students' Perceptions of the Environment.

	**M**	**SD**
**Pedagogical design**		
1. The learning objectives are clearly stated in each lesson.	3.98	0.80
2. The organization of each lesson is easy to follow.	3.84	0.76
3. The structure of the environment helps me focus on the learning.	3.56	0.72
4. Expectations of assignments are clearly stated.	3.93	0.83
5. Activities are planned carefully.	4	1.00
6. The content of the course worked well in a blended learning environment.	3.84	0.64
7. The presentation of the course content was clear.	3.84	0.75
Total	3.86	0.79
**Social design**		
8. I communicate with other students in this subject electronically.	3.86	0.80
9. I can ask my teacher what I do not understand.	3.47	1.01
10. I can ask other students what I do not understand.	3.79	0.78
11. Other students respond promptly to my requests for help.	4.07	0.65
12. The teachers gives me quick comments on my work.	4.09	0.91
13. My classmates and I regularly evaluate each other's work.	3.93	0.87
14. I was supported by a positive attitude from my teacher and my classmates.	4.07	0.58
Total	3.90	0.82
**Technical design**		
15. I can access the learning activities at times convenient to me	3.84	0.92
16. The online material is available at locations suitable for me	3.93	0.92
17. I am allowed to work at my own speed to achieve my leaning objectives	3.79	0.83
18. I decided how much I want to learn in a given period	3.63	0.79
19. I decide when I want to learn	4	0.97
20. Using blended learning allowed me to explore the interest of my own	3.18	0.68
Total	3.73	0.85

The students' positive attitude toward the pedagogical design was also revealed from the interview, in which they expressed their satisfaction with the design of tasks and contexts. For example, Student A expressed that the course was designed in the way that they needed to “find solutions to the problems” by themselves most of the time and he also enjoyed the discussions in class. Student C recognized the relative authentic contexts of the tasks, which helped her devote herself to the tasks. She mentioned that in learning to write a CV, the teacher asked the students to imagine the situation in which they were about to graduate and hunt a job. “I felt the topic was very relevant to me, so I was motivated to do this task well.” She told the interviewer.

Apart from the positive opinions, some students expressed their concern about the pedagogical design. For example, Student H said, “The online learning added to our workload. Sometimes I was scared of all the online assignments we had to finish after class.” And student G had difficulty adapting to this learning approach. “It seemed that we were learning by ourselves. I am not sure whether I have learned enough knowledge. I would rather learn how to write from the teacher.”

#### Students' Perception of Social Design

As seen from Table 5, the overall mean score of the social design was 3.90 (*M* = 0.82), indicating students' generally positive attitude toward the social design. The data gathered from the students' interviews also suggested that students were satisfied with the social design. For example, student B mentioned that she always received encouragement and help when dealing with difficult tasks. Item 11 (*M* = 4.07, SD = 0.65) (Other students respond promptly to my request), Item 12 (*M* = 4.09, SD = 0.91) (The teachers give me quick comments on my work) and Item 14 (*M* = 4.07, SD = 0.58) (I was supported by a positive attitude from my teacher and my classmates) scored higher than Item 9 (*M* = 3.47, SD = 1.01) (I can ask my teacher what I do not understand) and Item 10 (*M* = 3.79, SD = 0.78) (I can ask other classmates what I do not understand). This finding reveals that in the environment, both students and teachers responded to others promptly, but students had considerations when they needed to consult others.

When asked the reason for this, the students suggested that the teacher and the environment did provide them with the opportunity to seek help, but sometimes they felt reluctant to trouble others. Student E mentioned when he found something he failed to understand, he would prefer to figure it out by himself first and then seek help from the teacher and classmates. He told the interviewer: “I thought the teacher was busy, and my classmates were also busy, so I would prefer to figure it out by myself.”

#### Students' Perceptions of Technical Design

As for the technical design (see Table 5), the average score is 3.73 (SD = 0.85), which suggests that the environment provided relatively sufficient technological support to the students. Item 16 (*M* = 3.93, SD = 0.92) (The online material is available at locations suitable for me) and Item 19 (*M* = 4, SD = 0.97) (I decide when I want to learn) got higher scores, which indicates that students could enjoy the convenience of “anywhere” and “anytime” in the learning environment.

This positive attitude was demonstrated in the interview data collected from Student F who expressed his appreciation for the freedom and the sense of control brought by asynchronous discussion. He said, “I could finish the task at the time that is convenient for me as long as I did not miss the deadline. I like it.”

One thing worth noticing is that the mean score of Item 20 (Using blended learning allowed me to explore the interest of my own) is 3.18 (SD = 0.68), which falls toward the middle of the 1–5 scale. This score reveals that students did not think the resources of the blended learning environment play an important role in exploring their own areas of interest. In the interview, student D expressed that he did not find the resources very interesting, for the range of the topics was rather limited, and he was not attracted by the resources provided.

In sum, students' ratings on different dimensions of the questionnaire suggest that students perceived the productiveness of the learning environment in a generally positive way. This result is consistent with the studies exploring students' perceptions of the blending learning environment in general (e.g., Akkoyunlu and Yilmaz, 2006; Dziuban et al., 2006; Owston et al., 2006; Bendania, 2011; Wang and Huang, 2018). In the study conducted by Wang and Huang (2018), a blended environment was also constructed from the pedagogical, social, and technical perspectives. The findings of the study reveal that students are generally positive toward the design of the learning environment. This may suggest that students would perceive the learning environment positively if the elements of the blended learning environment are carefully designed. Despite the generally positive attitudes toward the learning environment, some students expressed their concern about the workload and adaptation to the way of learning in the interview. In study Stracke (2007), the way of learning was also found to make the students withdraw from the blended course. The findings indicate that some students may need more time to adapt to more student-centered learning.

### Students' Perceived Impact of the Blended Learning Environment on the Development of Their Critical Thinking

Drawing mainly on Paul and Elder's framework of thinking elements, the following themes emerged as to the students' perceived improvement of critical thinking after data analysis and are elucidated through students' quotations.

#### Gaining a Deeper Understanding of the Concept of “Critical Thinking”

In the interview, students talked about their improvement in understanding the concept of critical thinking. For example, Student D expressed that the environment helped him clarify the concept of critical thinking. He used to consider the concept as closely related to “criticizing” because of its Chinese translation and came to realize that it was closer to the concept of “rational thinking.”

Some students also expressed that the course helped them realize the importance of critical thinking. As the teacher clearly informed the students of the specific critical thinking skills each task aimed to cultivate, students realized that “critical thinking is not an abstract concept, but concrete ways of guiding people to solve problems” (Student B).

#### Using Facts and Evidence to Support One's Own Opinion

In the interview, students also talked about the change they experienced when forming and supporting their opinion. They started to recognize the importance of facts and evidence in their writing. Student E told the interviewer that he learned that supporting ideas were very important to make one's opinion accepted. He said: “In accomplishing the writing tasks of the course, I gradually learned to provide arguments with further explanations, examples and,… maybe some data.”

Some students also suggested that facts and evidence were important for them to convince others in the discussions. Student B said: “In the past when someone disagreed with me, I usually felt sad and angry. I would either remain silent or quarrel with them. In this course, I learned that if I wanted others to accept my opinion, I needed to convince them with evidence such as facts and information.” She also felt excited that her well-presented opinions were accepted several times during the discussion with her team members.

#### Thinking From Multiple Perspectives

Another perceived effect is thinking from multiple perspectives, which was mentioned by many students. For example, Student A described how a particular activity helped him recognize the importance of different perspectives and how his own writing benefited from a particular activity in the course. “The teacher asked some of us to play the role of employer and I was assigned this role. When I thought from the employer's perspective, I knew what kind of employee I needed… When I wrote my job application letter, I had a very clear idea what to include in my letter.” (Student A) Student F also mentioned that recognizing different perspectives helped him finish writing the complaint letter well. According to him, he not only mentioned the dissatisfaction in the complaint letter but also stated the potential negative impact on the company to which he sent the letter.

#### Exploring and Clarifying the Purpose Behind the Texts or Behaviors

The interviewees also mentioned that they learned to explore and clarify the purpose behind the texts or behaviors. Some students explained how they started to consider purpose as an important component in their writing. Student H told the interviewer that when the teacher started to teach a new genre, she always asked the students to discuss under what circumstances they could meet or use this type of writing, and why they needed it in the daily life. “In this way, I understand that there should be a clear purpose behind each writing. And… and when I tried to finish my own writing task, I also put the writing purpose into my consideration.” said Student H.

Some students also told the interviewer that they gradually learned to avoid distraction and stick to the purpose when they conducted a discussion. According to student G, the students tended to talk about irrelevant things when they had discussions at the beginning of the course. With the instructors' constant reminding, they could realize whether they strayed from the point and returned to the right track in time at the end of the semester.

In summary, the data from the interview suggest that students could perceive their critical thinking development in different thinking dimensions. Furthermore, according to the students' opinion, their development in critical thinking was also manifested in their writing and even transferred to other activities. As for the promoting factors of the development, the students recognized the importance of learning environment design, especially the pedagogical design and the social design. For example, students attributed their deeper understanding of the concept to the instructor's deliberate introduction of critical thinking and focus on the development of thinking skills in the activity design. Also, they believed that the teachers' guidance and peers' scaffold enabled them to realize the importance of multiple perspectives. These factors were also found to promote students' critical thinking in the systematic review conducted by Chou et al. (2018). This suggests that designing the elements of the learning environment to provide favorable conditions for critical thinking development could bring positive effects.

## Limitations and Implications

This study proposed the construction of a blended learning environment to promote critical thinking in terms of pedagogical, social, and technical design and explored students' perceptions of the environment design and their perceived impact on the improvement of critical thinking. The results of the study suggests that students are generally satisfied with the design of the learning environment, and students considered the learning environment helpful in improving critical thinking. Even though the study made a contribution to the instructional design aiming at critical thinking promotion in a blended learning environment, some limitations should be duly noted. First, because the participants of the study were 90 students in the same University, the relative homogeneity of the context may present a possible connection with the result. Therefore, replication is recommended with larger and more diverse samples. Second, the study was not able to present the relationship between environmental design and critical thinking development quantitively. Further study could focus on the correlation between design strategies and the improvement of specific thinking skills, or the predictive capability of elements design for the promotion of critical thinking.

This study also has some implications for critical thinking cultivation in the instruction of specific disciplines. On the one hand, the cultivation of students' critical thinking requires the detailed design of the blended learning environment. Special attention needs to be paid to pedagogical, social, and technical design covering factors such as learning objectives, student interaction, and ICT tools. On the other hand, students' troubles and challenges such as the extra workload and emotional factors should be taken into consideration when designing the learning environment.

## Data Availability Statement

The original contributions presented in the study are included in the article/supplementary material, further inquiries can be directed to the corresponding author/s.

## Ethics Statement

The studies involving human participants were reviewed and approved by School of Foreign Languages, Northeast Normal University. The patients/participants provided their written informed consent to participate in this study.

## Author Contributions

DL designed and implemented the learning environment, collected and analyzed the data, and wrote the article.

## Conflict of Interest

The author declares that the research was conducted in the absence of any commercial or financial relationships that could be construed as a potential conflict of interest.
